# Holiday Heart Syndrome in a 22-Year-Old Male: A Case Report

**DOI:** 10.7759/cureus.64106

**Published:** 2024-07-08

**Authors:** Nicholas L Todd, Shahriar Tahvilian

**Affiliations:** 1 Orthopedic Surgery, Aultman Hospital, Canton, USA

**Keywords:** cardiology, electrophysiology, alcohol use, atrial fibrillation, holiday heart syndrome

## Abstract

Holiday heart syndrome (HHS) is an acute cardiac arrhythmia following an episode of binge drinking. We report a healthy 22-year-old male with HHS after one night of binge drinking. The patient had no family history of cardiomyopathy, arrhythmia, or cardiac disease. Diagnosis was made after a thorough workup, including imaging and laboratory analysis.

After a two-year period with no repeat episodes of arrhythmia, the patient decided to follow up with electrophysiology on an as-needed basis. It is important for providers to consider HHS as a possible diagnosis of exclusion in cases of arrhythmia in the young population to avoid excessive healthcare costs.

## Introduction

Atrial fibrillation is one of the most common pathologies of the heart and one of the most frequent arrhythmias worldwide [1]. It generally affects older people in a population, although it can be seen in young adults (<45 years old), children, and adolescents [1]. The prevalence of atrial fibrillation is strongly age-dependent [2]. In those less than 40 years old, the prevalence is 0.5% compared to 5% in those older than 60 years old [2]. Arrhythmias such as atrial fibrillation that are caused by binge drinking have been called “holiday heart syndrome” (HHS) [3]. It was first described in the 1970s by Philip Ettinger, with the term HHS first being used in 1978 [3]. Ettinger et al. described the occurrence as an acute cardiac disturbance in otherwise healthy young people after an episode of heavy alcohol consumption [3]. Ettinger et al. noted that these events were more frequent following weekends and holidays, such as Christmas and New Year’s Eve, hence the name HHS [3]. Given the rarity of atrial fibrillation in young patients, we believe it is important to document cases so they may be further considered and not overlooked in this specific patient population. We describe an episode of HHS in a healthy 22-year-old male following an acute episode of binge drinking.

## Case presentation

A 22-year-old male college student with no past medical history presented to the emergency department (ED) with a chief complaint of his heart "not beating right." The patient reported he was consuming alcohol with his friends two nights prior to his presentation, including combined alcoholic and caffeinated beverages. He did not report any drug use and denied ever using drugs in the past. The day prior to his presentation, the patient reported the same feeling of his heart "not beating right" all day. The following morning, the patient presented to the ED after feeling lightheaded at the gym.

Vital signs were within normal limits except for moderately elevated blood pressure of 142/78. The physical exam was normal except for an irregularly irregular pulse. Chest X-ray was normal. EKG analysis demonstrated atrial fibrillation with a controlled rate of 80 beats per minute. Complete blood count, complete metabolic panel, urine drug screen, and thyroid cascade were all within normal limits at the ED. Cardiology was consulted and agreed that the patient could be discharged with 30 mg of metoprolol twice a day and 81 mg of aspirin daily. The aspirin was initiated out of caution due to the unknown etiology of the patient's arrhythmia at this time.

**Figure 1:**
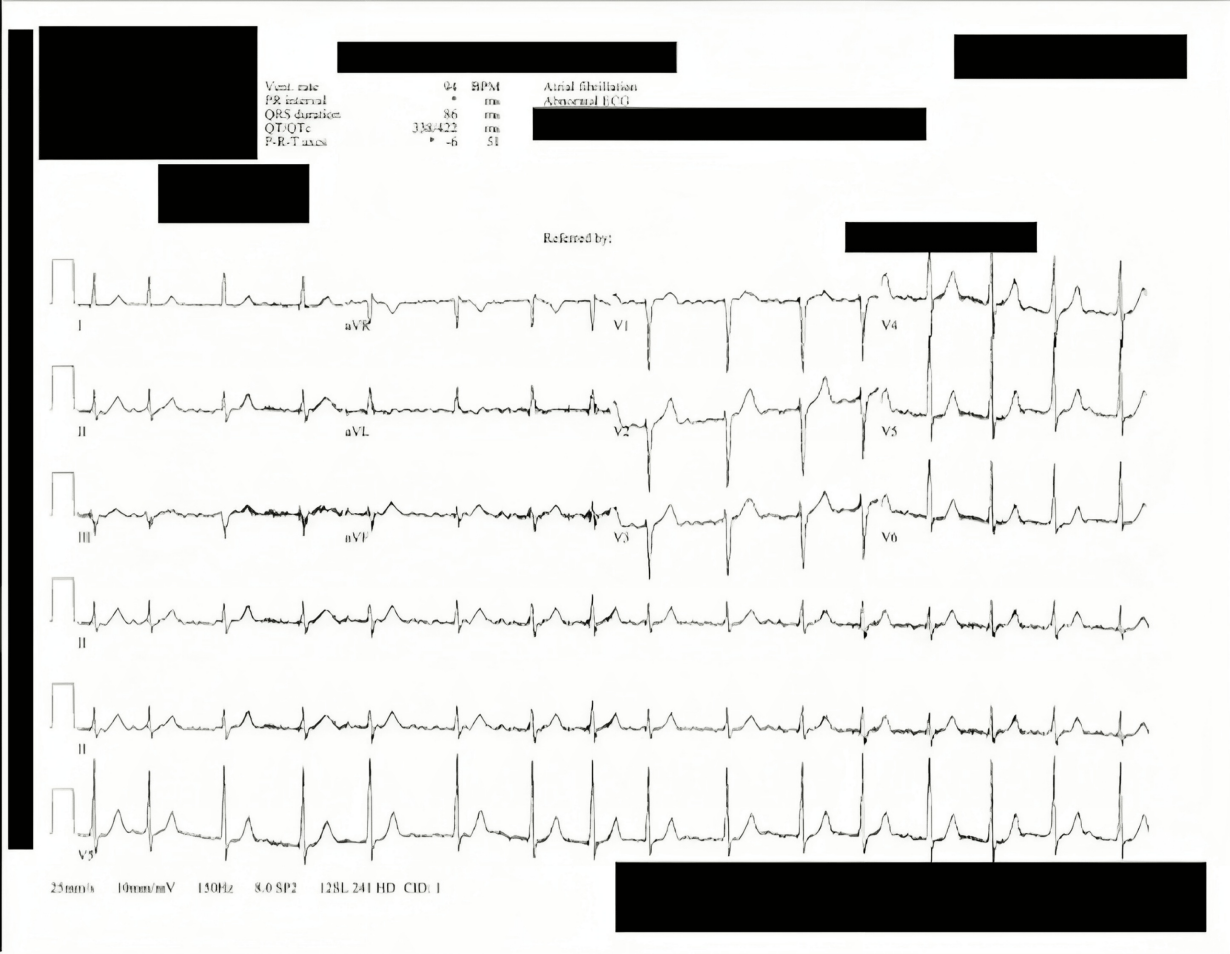
EKG at the cardiologist's office demonstrating atrial fibrillation, day 3 since ED visit. Personal health information is redacted ED: Emergency department

The patient followed up with cardiology three days after the initial presentation to the ED for an echocardiogram and repeat EKG. The echocardiogram was within normal limits; both the EKG and echocardiogram demonstrated atrial fibrillation at this time, at a rate of 94 beats per minute. The patient’s EKG at this visit is demonstrated in Figure 1.

The patient followed up with family medicine after one week to receive a full workup including thyroid-stimulating hormone (TSH), T3, T4, complete blood count, and complete metabolic panel. All were within normal limits. At the time of follow-up, the patient reported he felt like his heart was beating normally again. EKG at this time demonstrated normal sinus rhythm. Given an unknown exact time of atrial fibrillation onset, it was estimated that the patient experienced arrhythmia for about one week.

The patient sought a second opinion with electrophysiology at three months following the initial presentation. Repeat EKG at the electrophysiologist's office demonstrated normal sinus rhythm, repeat echocardiogram was normal. Metoprolol and aspirin were discontinued at this time.

The patient was scheduled for a repeat echocardiogram at seven months which demonstrated normal sinus rhythm. No structural or flow abnormalities were detected. The echocardiogram was repeated out of caution.

At two years of follow-up with electrophysiology, the patient reported no repeat episodes of atrial fibrillation. A diagnosis of HHS was made. Repeat thyroid cascade at a two-year follow-up with family medicine was within normal limits. The patient elected not to pursue further medicine workup in agreement with the electrophysiology diagnosis of exclusion. The patient did report a gradual return to caffeine and alcohol use but did not mix the substances. Two years since the initial ED visit, the patient decided to continue follow-up with electrophysiology on an as-needed basis.

## Discussion

Atrial fibrillation is uncommon in the young adult population [2]. In the young, it can be an initial presentation of genetic or structural pathology such as Brugada, long QT, Wolff-Parkinson-White (WPW) syndrome, or cardiomyopathy [1]. A thorough workup of atrial fibrillation in the young is therefore critical to explore possible electrical or structural pathology. HHS can be considered in patients with a clinical history of arrhythmia following binge drinking after more sinister causes for the disturbance have been ruled out. HHS is a diagnosis of exclusion. The most common arrhythmia from HHS is atrial fibrillation [4].

Management of atrial fibrillation in young adults is largely derived from that of older adults [1]. This includes beta blockers and the calculation of the CHADS-VASc score. In our patient, the score was calculated to be zero; however, he was still placed on 81 mg aspirin daily at the initial presentation out of an abundance of caution since the etiology of his arrhythmia was unknown at his initial presentation [5]. Cardioversion was considered as a possible initial treatment for our patient; however, given the unknown exact onset of atrial fibrillation and lack of immediate echocardiogram availability at the hospital, cardiology decided to not cardiovert at ED presentation. In line with adult recommendations, cardioversion can also be considered for young adults.

The atrial fibrillation caused by HHS usually terminates spontaneously within 24 hours [6]. About 26% of patients experienced a subsequent episode of HHS with alcohol binges within one year [6]. Habitual and binge drinking are both important risk factors for atrial fibrillation; however, it is currently unclear how to best manage an acute episode of HHS long-term, apart from abstinence from alcohol [4,6]. Cardiac arrest has been reported secondary to HHS [7].

The exact cause of HHS is not well understood, but it is thought to be related to the effects of alcohol on the heart and blood vessels. The effects of alcohol on the heart can either be direct through myotoxicity or indirect through its effects on other organs [3]. One theory suggests that HHS is caused by the possible slowing of the cardiac conduction system due to acute alcohol ingestion [3]. Cardy et al. demonstrated a slowing of P and QRS waves after acute alcohol consumption in 13 participants [8]. It is currently unclear how caffeine modulates the conduction system in conjunction with alcohol.

There is an emerging connection between the consumption of caffeine in energy drinks and the onset of atrial fibrillation in young patients [9]. Atrial fibrillation secondary to the overconsumption of caffeine has been demonstrated both with and without the addition of alcohol [9]. A total of 25% to 40% of young adults report energy drink use during parties with alcohol consumption, which may put them at an increased risk for possible HHS [10]. There are currently no studies regarding the association of HHS and caffeine specifically.

There are many unanswered questions regarding HHS. Some factors to be explored include the rate of alcohol ingestion in relation to HHS, if the type of beverage is associated with incidence, and if there is a genetic association with HHS. It is currently thought that the incidence of HHS is underestimated since atrial fibrillation can present with no symptoms at all; however, there are few studies regarding HHS incidence [3]. Another possible area of interest may include how caffeine and alcohol, in combination, modulate the cardiac conduction system to produce arrhythmia in HHS.

## Conclusions

HHS is a rare arrhythmia that occurs after alcohol consumption. A thorough workup is critical to rule out more sinister causes of arrhythmia, especially in the younger population.

HHS should be considered a diagnosis of exclusion in the initial workup of arrhythmia. The prognosis is good, as most arrhythmias spontaneously cardiovert to normal sinus rhythm within 24 hours and do not tend to recur with alcohol abstinence.

## References

[1] Gourraud JB, Khairy P, Abadir S (2018). Atrial fibrillation in young patients. Expert Rev Cardiovasc Ther.

[2] Sankaranarayanan R, Kirkwood G, Dibb K, Garratt CJ (2013). Comparison of atrial fibrillation in the young versus that in the elderly: a review. Cardiol Res Pract.

[3] Tonelo D, Providência R, Gonçalves L (2013). Holiday heart syndrome revisited after 34 years. Arq Bras Cardiol.

[4] Voskoboinik A, Prabhu S, Ling LH, Kalman JM, Kistler PM (2016). Alcohol and atrial fibrillation: a sobering review. J Am Coll Cardiol.

[5] Zhu WG, Xiong QM, Hong K (2015). Meta-analysis of CHADS2 versus CHA2DS2-VASc for predicting stroke and thromboembolism in atrial fibrillation patients independent of anticoagulation. Tex Heart Inst J.

[6] Krishnamoorthy S, Lip GY, Lane DA (2009). Alcohol and illicit drug use as precipitants of atrial fibrillation in young adults: a case series and literature review. Am J Med.

[7] Fuenmayor AJ, Fuenmayor AM (1997). Cardiac arrest following holiday heart syndrome. Int J Cardiol.

[8] Cardy MA, Donnerstein RL, Kelly LF, Bittner NH, Palombo GM, Goldberg SJ (1996). Acute effects of ethanol ingestion on signal-averaged electrocardiograms. Am J Cardiol.

[9] Mattioli AV, Pennella S, Farinetti A, Manenti A (2018). Energy drinks and atrial fibrillation in young adults. Clin Nutr.

[10] Bigard AX (2010). Risks of energy drinks in youths (Article in French). Arch Pediatr.

